# *Exophiala dermatitidis*: Key issues of an opportunistic fungal pathogen

**DOI:** 10.1080/21505594.2019.1596504

**Published:** 2019-04-03

**Authors:** Lisa Kirchhoff, Maike Olsowski, Peter-Michael Rath, Joerg Steinmann

**Affiliations:** a Institute of Medical Microbiology, Center of Excellence in Clinical and Laboratory Mycology and Clinical Studies, University Hospital Essen, University of Duisburg-Essen, Essen, Germany; b Institute of Clinical Hygiene, Medical Microbiology and Infectiology, Klinikum Nürnberg, Paracelsus Medical University, Nuremberg, Germany

**Keywords:** *Exophiala dermatitidis*, *Wangiella dermatitidis*, black yeast, cystic fibrosis, phaeohyphomycosis, polymorphism, virulence, pathogenicity

## Abstract

The black yeast *Exophiala dermatitidis* is an opportunistic pathogen, causing phaeohyphomycosis in immunosuppressed patients, chromoblastomycosis and fatal infections of the central nervous system in otherwise healthy Asian patients. In addition, it is also regularly isolated from respiratory samples from cystic fibrosis patients, with rates varying between 1% and 19%.

Melanin, as part of the cell wall of black yeasts, is one major factor known contributing to the pathogenicity of *E. dermatitidis* and increased resistance against host defense and anti-infective therapeutics. Further virulence factors, e.g. the capability to adhere to surfaces and to form biofilm were reported. A better understanding of the pathogenicity of *E. dermatitidis* is essential for the development of novel preventive and therapeutic strategies. In this review, the current knowledge of *E. dermatitidis* prevalence, clinical importance, diagnosis, microbiological characteristics, virulence attributes, susceptibility, and resistances as well as therapeutically strategies are discussed.

## Introduction

The incidence of fungal infections has increased in recent decades, posing new challenges to health care professionals [1]. One group of fungi found as pathogens in clinical specimen are the black yeasts, also known as black yeast-like fungi. These are mainly characterized by their dimorphic character, being able to switch from the yeast-like to the hyphal state. In addition, the dark appearance of colonies, caused by the melanized and thick cell wall is another main characteristic of black yeasts. This melanization represents one virulence attribute. However, not all black yeasts are known to be human pathogens, e.g. *Aureobasidium spp*. being plant-associated fungi [2]. In a clinical context, black yeasts are most frequently associated with the genus of *Exophiala*. The most prominent representative and at the same time most isolated melanized fungus is *Exophiala (Wangiella) dermatitidi*s, found as a pathogen in the human host. Next to *E. dermatitidis*, also other species of the genus known to infect humans, e.g. *E. oligosperma, E. jeanselmei and E. xenobiotica* [3]. The number of studies dealing with *E. dermatitidis* as a human pathogen has increased in recent years, highlighting the importance of *E. dermatitidis* in medical mycology. Among others, the black yeast-like fungus *E. dermatitidis* is a frequent colonizer of the respiratory tract of cystic fibrosis (CF) patients with varying rates of up to 19% [4–7]. In addition to exacerbation of CF, *E. dermatitidis* is also known to be a cause of central nervous system infections in otherwise healthy, immunocompetent Asian patients [8–11]. Furthermore, cases of *E. dermatitidis* infections in immunosuppressive patients have been reported, most often in the form of phaeohyphomycosis [12,13]. The fungus is globally prevalent and case reports on *E. dermatitidis* infections display the different groups of patients at risk. Various studies focused on virulence factors, e.g. pigmentation, polymorphism, hydrophobicity, adhesion and biofilm formation, production of secondary metabolites and so on, as well as its susceptibility against antifungal agents in both, clinical and environmental isolates. In this review, the current knowledge of *E. dermatitidis* prevalence, clinical importance, diagnosis, microbiological characteristics, virulence attributes, susceptibility, and resistances as well as therapeutically strategies are discussed.

**Figure 1.**
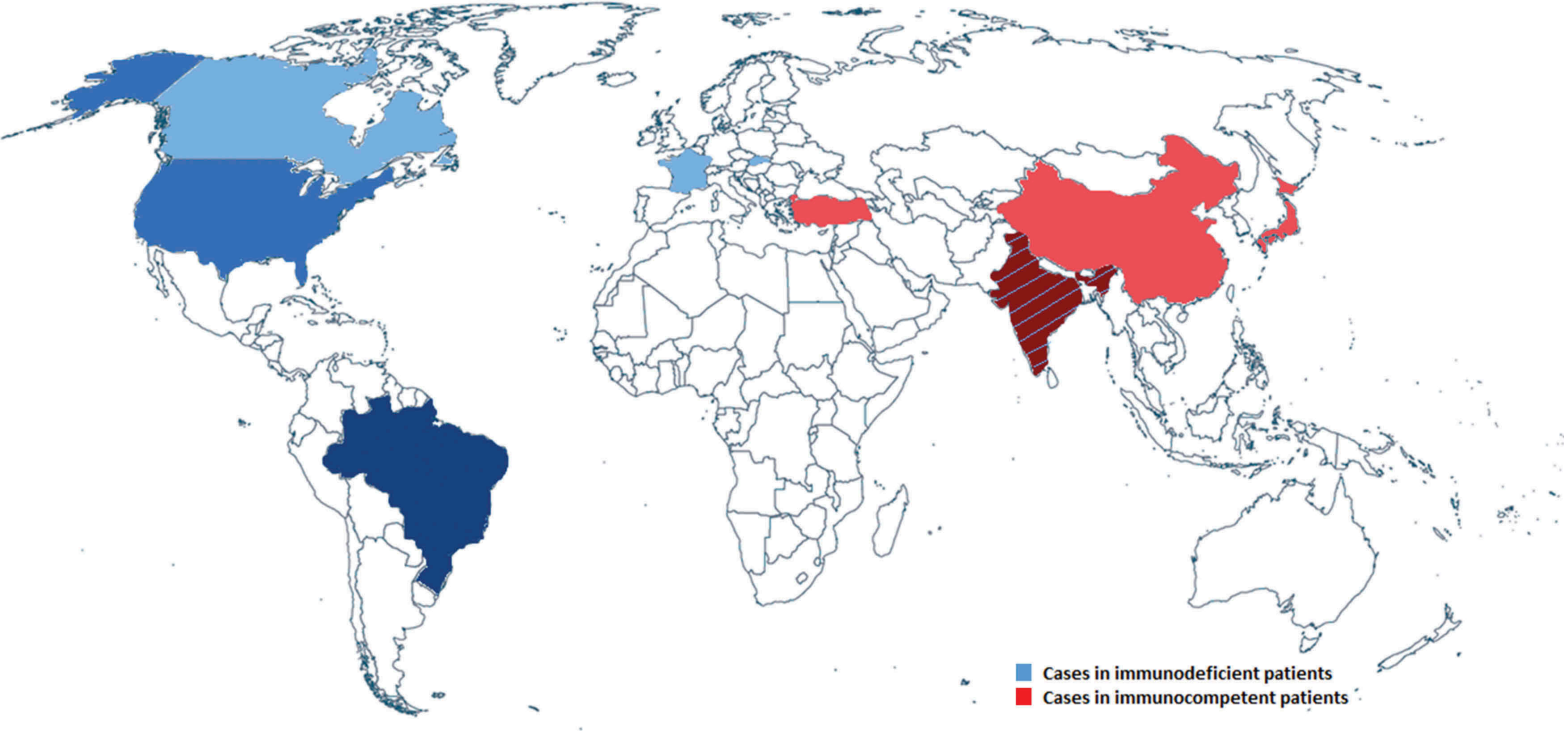
Prevalence of *E. dermatitidis* caused infections in immunocompetent (red = 9 cases) and immunodeficient (blue, 9 cases) patients as reported in case reports since 2007.

**Figure 2.**
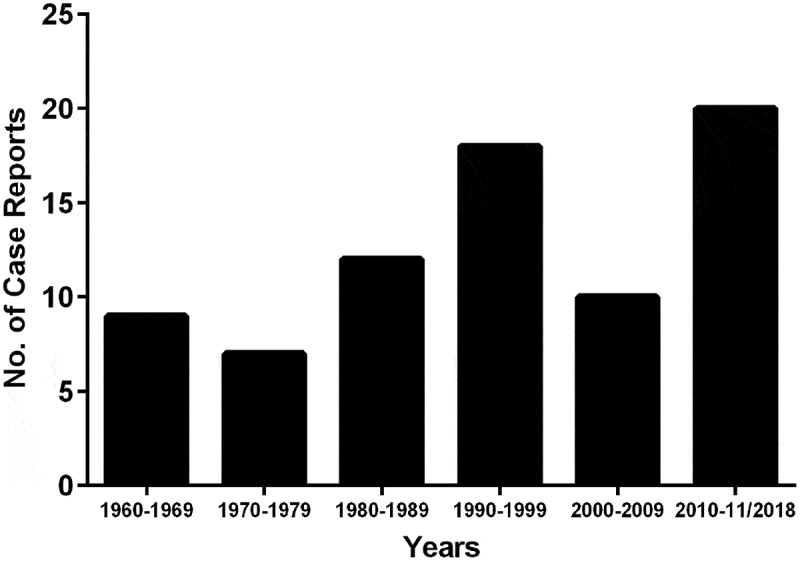
Number of cases of *E. dermatitidis* infections reported in the literature from 1960 to May 2018. Data until 1999 were retrieved from Taj-Aldeen et al. [12]. Latest reports were recorded by carefully scrutinized for case reports.

## Historical and clinical importance and today’s prevalence

*E. dermatitidis* was first isolated in 1937 by Kano from a lesion on the cheek of a Japanese woman and was classified as *Hormiscium dermatitidis* [14]. Due to its morphology, the taxonomic classification of *E. dermatitidis* has varied frequently. In view of the various sporulation patterns and the morphology of conidia and conidiophores, it has been classified in the genera *Fonsecaea, Hormodendrum, Phialophora, Rhinogladiella, Exophiala,* and *Wangiella* [14]. Today *Exophiala* as well as *Wangiella* are commonly used.

The taxonomy of black yeasts was confusing in the past. After the application of molecular criteria, a huge number of species belonging to the black yeasts was encountered in both, environmental and clinical settings [15]. The order *Chaetothyriales* with the family *Herpotrichiellaceae* comprises clinically relevant black yeasts and relatives [15]. One genus within this family is *Exophiala*. The genus *Exophiala*, characterized by annellidic conidiogenesis, currently comprises more than 40 species [16,17]. *Exophiala spp*. belong to the saprophytic fungi which can be isolated from hydrocarbon rich or warm, humid and oligotrophic environments [18]. Some species are solely producing budding cells, some also forming phialidic collarettes, sympodial conidiophores or conidial chains in various ratios [16,17,19]. Among the genus, *Exophiala* are the most clinically relevant black yeasts with reported mortality rates of 25–80% in systemic and invasive cases, even though fatal systemic cases are relatively rare [20,21]. In contrast, several *Exophiala* spp. are known to cause cutaneous and superficial infections in human and animals [3,19]. In a study regarding the spectrum of clinically relevant *Exophiala* species in the US, 188 strains, previously identified as *Exophiala* ssp., were investigated. In the following, all species identified with rates above 3% are listed: *E. xenobiotica* (19.7 %), *E. oligosperma* (18.6%), *E. lecanii-corni* (6.9 %), *E. phaeomuriformis* (6.4%), *E. jeanselmei* (3.7%), *E. bergeri* (3.7%), and *E. mesophila* (3.2%) [3]. Systemic infections were mainly caused by *E. dermatitidis*, which is in addition also the most frequently isolated *Exophiala* species (29.3%) [3].

*E. dermatitidis* is a ubiquitous fungus, although rarely isolated from the environment [18]. It is assumed that the origin of this fungus lies in the tropical rainforests, among others in the niche of wild fruits and berries. A possible route of infection by dispersal via feces from frugivorous birds and bats is reported by Sudhadham et al. This group investigated the origin of over 3,000 samples within a period of 3 y. In contrast, the prevalence in soil and plants was almost zero [18]. Little is known about the natural habitat and the transmission routes of *E. dermatitidis*. A recent review excellently summarizes the ecology of this fungus and reported that it can be mostly found in man-made indoor habitats, connected to water sources [22]. In the man-made environment, the fungus is globally distributed, found in dishwashers, steam baths or sauna facilities, all of those environments are characterized by high temperatures, humidity and pH changes [9,10,22–26]. In subtropical regions, *E. dermatitidis* was isolated from railway sleepers at a rate of 13% [27]. Due to the high isolation rate of *E. dermatitidis* in wastewater samples from dishwashers, it is posited that the dishwasher is a possible transmission route between environment and the human host, especially through aerosol inhalation [22]. However, household-acquired infections seem to be rare and either mild [28] or restricted to patient populations with disorders such as CF [22] or immunosuppression [29].

No relevant hints exist that *E. dermatitidis* would be transmitted via a zoonotic route. Two cases reported invasive *E. dermatitidis* (intraabdominal and subcutaneous) in dogs [30,31] and in an older study *E. dermatitidis* was found in the liver of fruit-eating bats, *Eidolon helvum* [32].

### E. dermatitidis *in cystic fibrosis (CF)*

*E. dermatitidis* is, among other species, a common colonizer of the respiratory tract of patients with CF. In 1990, Haase et al. [7] reported for the first time *E. dermatitidis* isolation from the sputum of a 5-year-old CF patient, even after treatment with amphotericin B and 5-fluorcytosine. In 1992, Kusenbach et al. [33] described an *E. dermatitidis-*associated pneumonia in a 7-year-old patient with CF. The isolation rates of *E. dermatitidis* varied in numerous studies between 1% and 19% in CF [4–7]. The prevalence of *E. dermatitidis* in Germany, Sweden, and Belgium is the highest in the world with rates ranging from 4.8% to 17% in contrast to lower rates in other countries like France or the USA [5,34]. The varying rates of *E. dermatitidis* isolation may be correlated to genetic factors, as described for *P. aeruginosa*, or to lifestyle variations [34]. Another important factor is the existence of variances in isolation success due to lack of standardization in the mycological analysis of respiratory material [34], as well as problems in the identification of *E. dermatitidis*, e.g. due to failure in distinguishing between different *Exophiala* spp. Reliable diagnostic methods for *E. dermatitidis* identification are mentioned in the section “Diagnosis of infections.”

Lebecque et al. described a prevalence of *E. dermatitidis* to colonize the respiratory tract of pancreatic insufficient CF patients [6]. Additionally, predisposing factors for *E. dermatitidis* infections are diabetes mellitus, steroid medication, concurrent bacterial and fungal infections and nutritional deficiencies [14]. In addition, most *E. dermatitidis* isolates from CF patients occurred in adolescent or adult patients [6]. Grenouillet et al., in 2018 [35], described two cases of mild forms of CF identified in elderly patients following a diagnosis of a respiratory infection or colonization by *E. dermatitidis*. It was suggested that in patients with chronic respiratory disease and recurrent pulmonary infections, the detection of *E. dermatitidis* could be a potential marker of atypical CF and should lead clinicians to conduct investigations for CF diagnosis [35].

Traditionally, *E. dermatitidis* is considered to have low virulence in CF patients [14]. The differentiation between colonization and infection with *E. dermatitidis* in a CF patient is analog to relevant CF bacteria, e.g. *Staphylococcus aureus* or *Pseudomonas aeruginosa* very difficult. However, most CF patients show no clinical symptoms. In patients with pulmonary exacerbation and/or decline in lung function, the clinical significance of *E. dermatitidis* can be evaluated with the following diagnostic parameters: despite the culture positivity it is possible to analyze specific *E. dermatitidis* IgG antibodies. A recent work from Sweden reported that 4 of 17 CF patients had a symptomatic *E. dermatitidis* infection and showed clinical response to antifungal treatment [5]. It was shown that increased IgG serum levels against *E. dermatitidis* were positively associated with higher white blood cell counts, increased erythrocyte sedimentation rate, pancreatic insufficiency, antibiotic treatment and were negatively associated with respiratory function (FEV1% predicted) [5]. Specific IgG detection is not commercially available so far. The fungal biomarker 1,3-ß-D-glucan can be easily determined in serum. Higher glucan levels were associated with positive serum IgG levels against *E. dermatitidis* [5]. If no other reasons could be identified for clinical deterioration in CF patients with repeated *E. dermatitidis* isolation in the airways, the initiation of antifungal treatment might be the last way to evaluate if the patient’s status improves. No larger studies exist that have investigated whether *E. dermatitidis* contributes to the disease outcome of CF patients. Therefore, it remains elusive whether *E. dermatitidis* is actively involved in CF lung disease pathologies or whether it rather reflects a dysregulated airway colonization and acts as a microbial bystander [5].

### E. dermatitidis *causing phaeohyphomycosis in immunosuppressed patients and fatal infections in immunocompetent individuals*

In addition to CF patients, also immunosuppressed or elderly patients as well as immunocompetent patients with Asian background are known to be affected by *E. dermatitidis*. The prevalence of *E. dermatitidis* infections in humans is global with significantly increased cases of infected immunocompetent patients in Asia. Also, infected immunodeficient patients are globally distributed. Risk of infection in those patients are immune defects like CARD9 mutations, immunodeficiency caused by drug treatment, cancer or transplantation [36–38]. Case reports of *E. dermatitidis* infections, occurred since 2007, are visualized in a map of the world with infections of immunosufficient patients indicated by blue color and immunocompetent infected patients indicated by red color (Figure 1). CF case reports were not included.

Immunosuppressed and elderly patients suffer from infections with *E. dermatitidis* most commonly in the form of phaeohyphomycosis, keratitis or chromoblastomycosis; 54 cases of phaeohyphomycosis have been described between 1934 and 2006 (Figure 2) [12,13]. Literature research on recent published case reports involving *E. dermatitidis-*caused infections revealed 20 new cases since 2007 in both, immunocompetent and -suppressed patients (Table 1) [21,36–51]. *E. dermatitidis* may also be a severe agent of pneumonia [12]. *Ex-vivo* skin-model evidence of *E. dermatitidis* penetration suggests that *E. dermatitidis* may also be responsible for superficial skin infections [52].

**Table 1.** Summary of *E. dermatitidis* caused infections case reports since 2007.

Age (gender)	Underlying medical condition	Immunodeficiency status	Possible route of infection	Geographical region (Back ground)	Clinical manifestation	Year	Ref
81 (f)	Localized bronchiectasis	Competent	Unknown	Japan	Bronchial infection	2007	[48]
24 (f)	Tinea versicolor for skin lesion	Competent	Traumatic inoculation	Turkey	Sclerosing cholangitis	2009	[49]
3 (m)	Non	Competent	Hematogenous	China	CNS infection	2009	[50]
8 (m)	Non	Competent	Unknown	Turkey	Systemic phaeohyphomycosis	2009	[51]
65 (m)	Hypertension, Multiple myeloma	Competent	Unknown	Japan	Lung nodule	2012	[22]
40 (f)	Kidney transplant	Compromised	Possibility of primary subcutaneous infection	India	Endocarditis	2013	[52]
60 (m)	Chronic herpes zoster keratitis	Unknown	Unknown	USA	Endophthalmitis	2014	[53]
21 (m)	Left ear abscesses	Competent	Direct extension to the brain	India	CNS infection	2014	[41]
57 (m)	Graft-versus-host disease, Mantle cell lymphoma, Hematopoietic stem cell transplant	Compromised	Unknown	USA	Fungemia	2014	[40]
8 (f)	Card9 deficiency	Compromised	Unknown	France (Nigeria)	Liver and brain infection	2015	[38]
78 (m)	Non	Competent	Unknown	China	Ulcer on right forearm	2016	[42]
8 (m)	Allogenic stem cell transplantation, acute myeloid leukemia	Compromised	Unknown	Slovakia	Systemic phaeohyphomycosis	2017	[43]
48 (f)	Lepromatous leprosy	Compromised	Unknown	Brazil	Subcutaneous phaeohyphomycosis	2017	[44]
64 (m)	Kidney transplant	Compromised	Unknown	Brazil	Pneumonia	2017	[39]
14 (f)	Ewing’s sarcoma	Compromised	Unknown	Brazil	Fungemia, disseminated infection	2017	[39]
44 (f)	Hip fracture	Unknown	Unknown	Brazil	Fungemia, disseminated infection	2017	[39]
3 (m)	Soft tissue sarcoma	Compromised	Unknown	Brazil	Fungemia, disseminated infection	2017	[39]
15 (f)	Non	Competent	Unknown	India	Phaeohyphomycosis	2017	[45]
28 (f)	Unknown	Compromised	Inoculation through skin	Canada (India)	Osteomyelitis, septic arthritis	2018	[46]
59 (m)	Uneventful cataract surgery	Competent	Unknown	India	Endolphtalmitis	2018	[47]

Fatal brain infections caused by the neurotropic *E. dermatitidis* occurred in otherwise healthy individuals in the Asian population [8–11]. Its neurotropic character has been hypothesized to be caused in parts by the capability to assimilate aromatic (alkylbenzene) hydrocarbons [53] and by the capability of cell–cell and cell-surface adherence [19]. Several studies revealed a predilection of *E. dermatitidis* for the human central nervous system. These infections are chronic but deaths attributed to them are only reported in Asia [10,54]. The restriction to Asian populations of cerebral *E. dermatitidis* infection cases is currently not sufficiently explained. Hypotheses are, among others the possibility of unequal exposure to the fungus [10] and involvement of different (immunological) host factors [54,55].

Outside Asia, nosocomial-acquired infections in pseudoepidemic situations have been reported, for example from the US. These infections mostly appear after administration of contaminated medication to elderly people [56,57]. Recently, 15 cases of *E. dermatitidis* bloodstream infections occurred among patients which were treated in a hemato-oncological hospital [58]. A contaminated intravenous flush solution was identified as the source of infection. These infections posed a challenge to clinical management of bloodstream infections with *E. dermatitidis*, as they are relatively rare and no specific guidelines for treatment of these infections exist [58].

### *Diagnosis of* E. dermatitidis *infections*

The diagnosis of infections with black yeasts is a challenge in routine diagnostics. Invasive fungal infections diagnosed delayed and appropriate delayed treatment may worsen the patients' outcome [59]. Thus, appropriate methods for species-specific identification are necessary. However, no species-specific standardized diagnostic tools are available.

Identification as *E. dermatitidis* by cultural methods, microscopy, and ribosomal DNA internal transcribed spacer (ITS) sequencing are commonly used techniques [21]. Morphological identification of *E. dermatitidis* is restricted by its slow-growing behavior [29] and therefore the risk of bacterial overgrowth, e.g. by *P. aeruginosa* and *Burkholderia cepacia* complex organisms, especially in CF patients [60]. Sabouraud agar is the most common used agar for isolation and cultivation of fungal species. However, *E. dermatitidis* isolation is recommended to be either performed on *Burkholderia cepacia*- selective agar, potato-dextrose agar with rose-bengal and chloramphenicol, erythritol-chloramphenicol agar (ECA) or Sabouraud gentamicin-chloramphenicol agar (SGCA), all leading to increased isolation rates compared to Sabouraud media by inhibiting bacterial growth [6,29,60–62]. The median time necessary for isolation was ascertained to be at least 6 d using ECA and SGCA [6]. As reported by Horré et al., prior to the use of ECA, *E. dermatitidis* was never isolated in routine work [61]. However, even when bacterial overgrowth is prevented, microscopic and macroscopic morphological identification on species level of black yeast may not be the optimal method. Often, species-specific morphology is absent/difficult to detect or variable expression of characteristics make it impossible to distinguish between species [63,64]. In addition, patients with invasive fungal infections may nevertheless be culture negative. Thus, alternative diagnostic tools should be considered.

Matrix-assisted laser desorption-ionization time of flight mass spectrometry (MALDI-TOF) has been proven to be an optimal method for rapid identification of pathogenic yeast from cultures [65,66]. Also, the identification of yeast belonging to the genus *Exophiala* by MALDI-TOF MS showed consistency with ITS sequencing analysis [67]. However, for distinguishing between species of the black yeast, robust reference spectra for every species are necessary [68,69].

Molecular methods are more and more replacing morphology tools for identifying fungi. A species-specific PCR introduced by Nagano et al. in 2008 was the first successful approach for a diagnostic *E. dermatitidis* PCR, based on rDNA operons and ITS regions[70]. Also, others suggest discriminative ITS data as the method of choice for species identification [63]. The ITS region is a barcode marker for the identification of fungi and ITS analysis has been shown to be a useful tool for distinguishing black yeasts [71,72]. This technique is even used to distinguish between *E. dermatitidis* subtypes [72]. However, initial studies did not demonstrate an advantage of molecular identification techniques over conventional cultivation techniques using an appropriate medium [73]. A multi-diagnostic approach including two or more methods should be considered for a reliable black yeast diagnostic.

## Diversity

*E. dermatitidis* diversity was analyzed using pheno- and genotypic methods. One phenotypic tool for discrimination and diversity analysis is fatty acid methyl ester (FAME) profile analysis [74]. Genotypic characterization of *E. dermatitidis* strains was among others performed using ribotyping of the small subunit of rDNA and by random amplification of polymorphic DNA (RAPD) [55,74], as well as by the commonly used analysis of the ITS1. Ribotyping resulted in differentiation of 21 clinical isolates, originating from CF sputum samples and systemic neurotropic infections in Asian patients, into two genotypic groups whereas RAPD analysis resulted in seven genotypes. The interpretation of these RAPD analyses is limited as the number of primers used may skew results and interlaboratory reproducibility of RAPD patterns is low [74].

Three main genotypes of *E. dermatitidis* have been detected in sequence analysis of the ITS1. These are denoted as groups A, B, and C [75]. Group A mostly corresponds to strains isolated from clinical specimens, showing a high frequency in virulence potential. Genotype B is mostly found in isolates from the natural environment, whereas isolates from the man-made environment occur in both genotypic groups, A and B, with slight predominance in the cluster of genotype A [75,76]. This genotypic cluster distribution is reported worldwide, indicating long-distance dispersal, especially for strains isolated from systemic infections [75]. Genotype A is most frequently identified [24,77]. In contrast, genotype C is rarely found.

## Microbiology & characteristics

**Figure 3.**
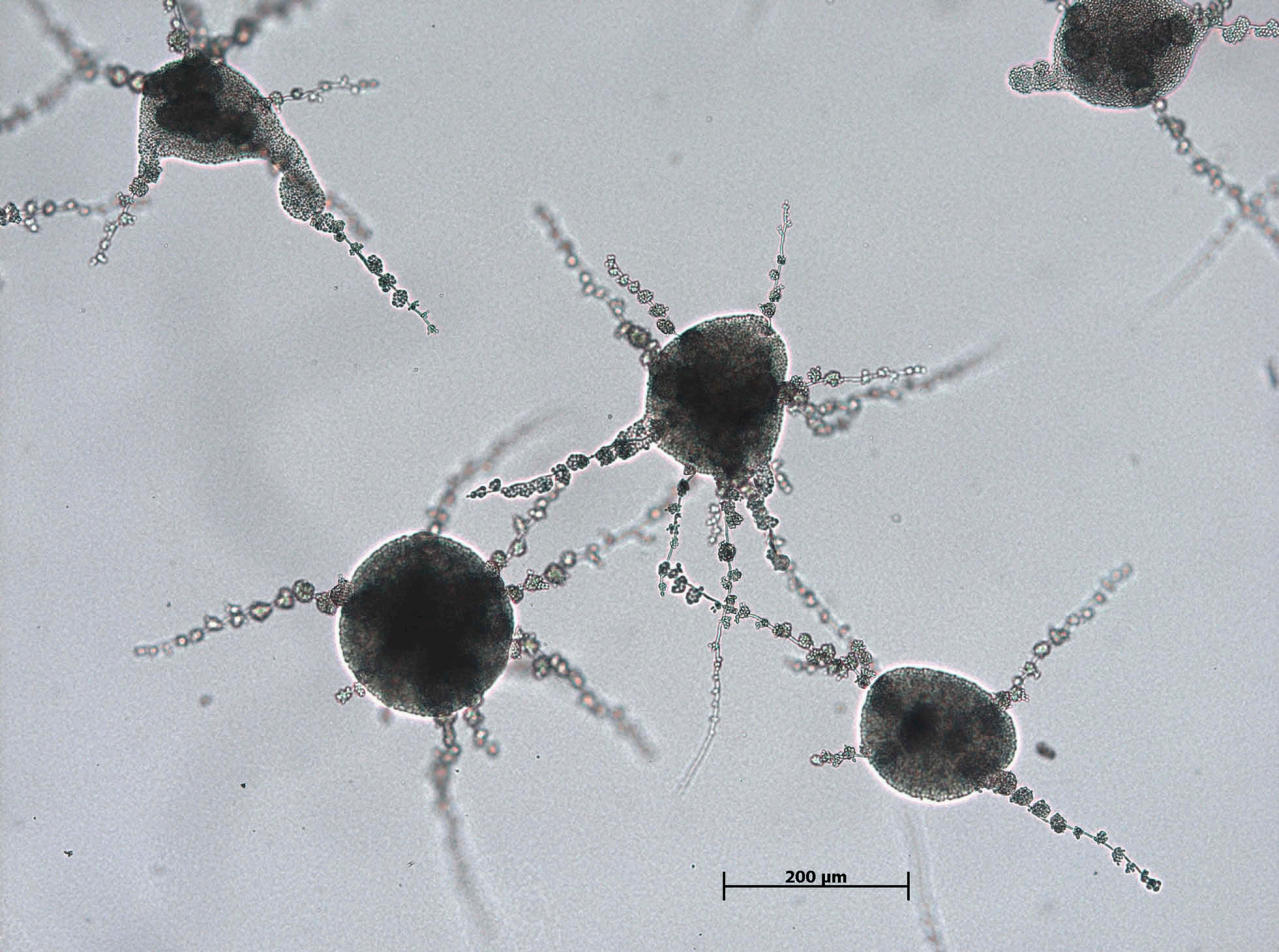
Light microscopy *E. dermatitidis* (CBS 149.90) colonies on malt extract agar after 48 h of incubation at 35°C. Scale bar equals 200 µm (Credits: M. Olsowski).

The black yeast-like fungus *E. dermatitidis* is dark-pigmented. Its morphology is from dimorphic character, filament and yeast forms are clearly visible in fungus microscopy (Figure 3). *E. dermatitidis* is currently accepted to be asexual as no sexual form has been discovered [78]. However, a phylogenetic analysis of various *Exophiala* species revealed a clade of the genus *Exophiala* with *Caproni mansonii*, a telemorph species, thus indicating *E. dermatitidis* being the anamorph of *C. mansonii* [17,79]. It is polyextremophilic, metabolically active over a wide temperature (4–40°C) and pH range (pH 2.5–12.5) [9,26,80–82]. *E. dermatitidis* is a slow-growing organism [29]. It is also known to be strongly hydrophilic [83]. In addition, black yeasts are characterized by the expression of specific gene families, such as alcohol and aldehyde dehydrogenase, membrane transporter proteins and cytochrome P450 [84]. Colonies of *E. dermatitidis* grow restricted, smooth, waxy and with a dark appearance, often with dark pigments exuded into the growth medium [17].

*E. dermatitidis* was identified to be a producer of exopolysaccharide (EPS), producing irregular EPS with a fibrillary substructure [2]. These EPS are associated with the fungus thermotolerance and thus with its virulence [2]. Possible roles of EPS in, e.g. biofilm formation, drug resistance, and immune evasion have been suggested as described for other fungi [85].

Among other factors, polymorphism is known to contribute to the virulence of certain fungi, as reported for thermally dimorphic species [86]. Also, *E. dermatitidis* ability to switch from the hyphal to yeast-like growth is associated with pathogenicity [17]. The transcription factor APSES, well known to regulate fungal cellular development and differentiation, is encoded by Wd*STUAp*. This transcription factor was shown to be a regulator of morphology in the dimorphic *E. dermatitidis* in both a negative and positive fashion [87].

Budding yeast cells are the predominant morphotype [87]. Conidia with a size of 2.5–4 × 2–3 µm are flask shaped and appear in small groups, free or intercalary [17]. Along with budding yeast cells, also pseudohyphal, moniliform hyphae, true hyphae, and sclerotic forms also appear. Yeast-to-hyphal transition has been monitored using electron microscopy [88]. Budding yeasts in their exponential growth phase were described to have thin cell walls. Aged cells, in contrast, showed altered cell structures with thick cell walls and a considerable amount of stored materials. Only the thick-walled cells were found to be capable of converting to hyphal structures, as the acquisition of “spore-like” characteristics (thick-walled, endogenous substrate reserves) is necessary for transition [88].

The polymorphism of *E. dermatitidis* was found to be calcium ion-dependent. A low calcium ion concentration results in non-polarized growth leading to multicellular form development in contrast to polarized growth and yeast budding or pseudo/true hyphal growth [89]. Similarly, temperature influences the form of the fungus, it being more likely to propagate in the filamentous form at room temperature, but switching to yeast-like growth at 37°C [90]. Sclerotic morphotypes can further be induced by nutrient-rich media at pH 2.5 [87].

## Virulence

**Figure 4.**
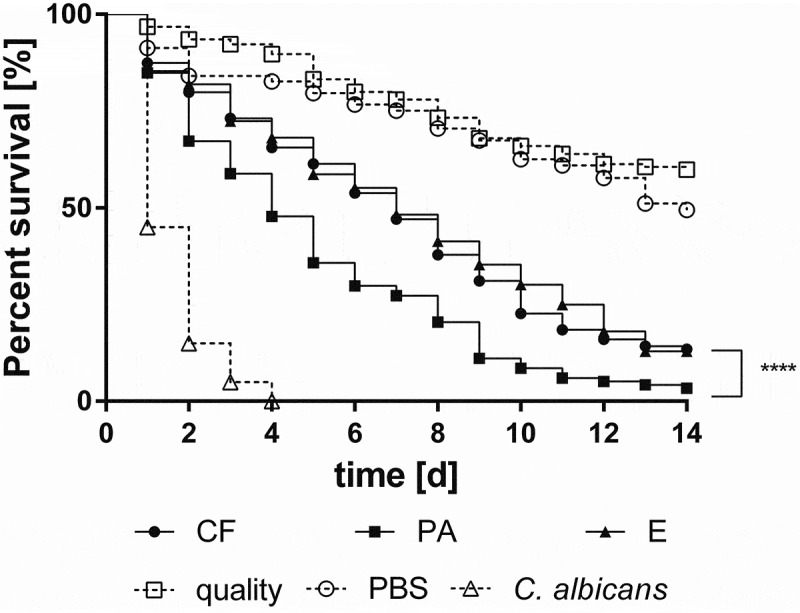
Survival curve of *Galleria mellonella* infected with *E. dermatitidis* Environmental isolates (E), CF patients isolates (CF) and Isolates from Asian, immunocompetent patients (PA). Depicted values are means of nine tested strains. Each strain was tested in triplicate. Taken from: Olsowski et al., 2018 [91].

*E. dermatitidis* showed virulence in *Galleria mellonella* [20,91] as well as in the nematode *Caenorhabditis elegans* [91]. The virulence of *E. dermatitidis* is furthermore dependent on the source of isolation as detected in a *G. mellonella* infection model (Figure 4). Dimorphism was identified as a key factor for the development of invasive hyphal growth and increased virulence, mostly showed by *E. dermatitidis* isolated from phaeohyphomycosis in immunocompetent Asian patients [91].

Additional to the above-described dimorphism of *E. dermatitidis* other virulence factors exist. The most prominent characteristic contributing to virulence is pigmentation [92–94]. Alongside melanin, carotenoids are also found in cell walls of black yeasts [95].

Capsular material is also a key determinant of virulence as it contributes to adhesion, phagocytose impairment and hindrance to complement-mediated killing [84]. The extracellular polysaccharides (acid mucopolysaccharides) secreted by *E. dermatitidis* yeast cells have been shown to mask the cells for human phagocytes during the invasion of tissue [2]. Nishimura and Miyaji suggested an additional role of extracellular polysaccharides in the interaction between yeast cells and mononuclear neutrophils in mice [25].

In addition, adherence and biofilm formation have been shown to contribute to virulence. Further virulence attributes of *E. dermatitidis* are hydrophobicity [96], urease, catalase, proteinase and DNase production [97], chitin synthase [98–100], secondary metabolite production [96,101] and the assimilation of aromatic (alkylbenzene) hydrocarbons [53,102,103]. Latter contributes to neurotropism characteristics of members of *Herpotrichiellaceae* [53]. The known virulence factors occurring in *E. dermatitidis* are summarized in Table 2.

**Table 2.** Virulence factors of *Exophiala dermatitidis.*

Virulence factor	Reference
Pigmentation	Dixon et al., 1987 [93]; Feng et al., 2001 [94]; Langfelder et al., 2003 [95]
Polymorphism	De Hoog & Guarro, 1995 [18]; Boral et al., 2018 [85],
Hydrophobicity	De Hoog, 1993 [97]
Biofilm formation and adhesion	Seneviratne et al., 2015 [131]; Sav et al., 2016 [98], Kirchhoff et al., 2017 [130],
Urease production	Sav 2016 [98]
Catalase production	Sav 2016 [98]
Proteinase (strain-dependent)	Sav 2016 [98]
DNase (strain-dependent)	Sav 2016 [98]
Encapsulation	Yurlova & De Hoog, 2002 [2]; Boral et al., 2018 [85]
Chitin synthases	Wang et al., 2001 [101]; Wang & Szaniszlo, 2002 [100]; Abramczyk & Szaniszlo, 2009 [99]
Assimilation of aromatic hydrocarbons	Prenafeta-Boldú et al., 2006 [55]; Isola et al., 2013 [103]
Secondary metabolite production	De Hoog, 1993 [97]; Kindler et al., 2010 [102]

### Pigmentation

One of the best-studied virulence factors of black yeasts and especially *E. dermatitidis* is the pigmentation, mostly associated with the pigment melanin [104].

Melanin is ubiquitous and found in all kingdoms of life and therefore also in many microbes (pro- and eukaryotes) as well as animals. The production of melanin in fungi has been documented since the early 1960s [94,105,106]. The melanin group of pigments comprises numerous and diverse substances, sharing a number of properties. They are all negatively charged, hydrophobic and high molecular weight compounds [106]. In the past, they were defined by their dark color, poor solubility and resistance to hot acids, hot and concentrated alkaline solutions and oxidizing acids [107]. Pigmentation of the cell wall of yeast is mostly associated with stability and protection due to the pigments structure, making it an extremely stable molecule. Pigments are able to protect the organism against biotic and abiotic stress factors, e.g. temperatures, osmotic pressures, UV light, radiation, and host immune-cells [108]. Additionally, in *E. dermatitidis*, the multilayered, melanin carrying cell wall plays a role in cell protection, prevents desiccation, functions as an antioxidant and is able to harvest metabolic energy [84,106,109–111]. The latter is explained by the fact that exposure of ionizing radiation to melanized fungi, e.g. *E. dermatitidis* showed an increase in growth and biomass by altered electronic properties of the cell wall stored melanin [111]. Dadachova *et al*. assumed that the capability of melanin to capture electromagnetic radiation and its oxidation-reduction properties contribute to the gain of metabolic energy in melanized fungi [112,113].

The general clinical significance of melanized fungi is considered as relatively low with a detection rate of approximately 10% in fungal isolates in the diagnostic microbiology lab. However, they are significant agents of phaeohyphomycosis developing after traumatic infections [19]. Despite their rarity in clinics, the melanized fungi became significant due to their occurrence and pathogenicity in immunocompromised patients. The rarity of invasive fungal infections in immunocompetent patients might be due to various cells of the host defense. Important cells for the defense against fungal pathogens are, e.g. dendritic cells, neutrophils, macrophages, NK cells, CD4 T cells (Th1, Th2, Th9, Th17, and Treg), and CD8 cells [19,114]. All those cells produce cytokines, reactive oxygen intermediates or antimicrobial peptides, contributing to host protection against fungal pathogens [114]. In contrast, immunosuppressive patients have in one or the other way a lack of those immune cells, leading to a higher risk of infections with invasive fungal pathogens.

Melanin synthesis in fungi is in most cases related to phenol oxidase activity. The phenoloxidase system has been discovered in numerous fungi, e.g. *Cryptococcus neoformans* and is composed of soluble enzymes with broad substrate specificity [115]. In addition, the substrate-specific pentaketide pathway for melanin synthesis is significant for certain developmental stages [115]. Two prominent melanins, 1,8-dihydroxynapthalene (DHN) melanin and l-3,4-dihydroxyphenylalanine (DOPA) melanin, named after intermediates of the synthesis pathway used, are known to contribute to the pathogenicity of certain fungi [94]. Some fungi are even capable to synthesize melanin in various pathways, e.g. *A. fumigatus* which is, additionally to DHN melanin production, able to synthesize the so-called pyomelanin in presence of L-tyrosine [116]. The pentaketide pathway for melanin synthesis was detected in *E. dermatitidis* and melanin produced by *E. dermatitidis* has been identified as DHN melanin [115]. The pentaketide pathway in *E. dermatitidis* can be successfully blocked by the addition of the pathway inhibitor tricyclazole [117]. The responsible genes for all three pathways, DHN, DOPA, and L-tyrosine, have been identified in the *E. dermatitidis* genome [95,118].

The production of DHN melanin has been associated with virulence due to the observations of melanin-deficient strains of *E. dermatitidis* and *C. neoformans* exhibiting a decreased virulence [92,93,119]. The considerable effect of melanin on the survival and resistance of the cell is mainly due to its protective role against both environmental stress as described above and the oxidants of host effector cells [104]. Melanin allows the fungus to escape phagocytosis and protects against free radicals by acting as a potent free radical scavenger, protecting the cell against oxidants generated by the immune effector cells of the host organism [19]. It also provides protection from damage by UV-light, temperature and salt extremes, antimicrobial drugs and peptides. Although melanin plays a role in evasion of oxidative stress, this function is mainly carried out by detoxifying enzymes [90,120]. Melanin might also play an immunosuppressive role during fungal infection as it was shown that melanin suppresses the production of proinflammatory cytokines [121,122].

Feng *et al*. analyzed the virulence of *E. dermatitidis* wild-type strains against melanin-deficient mutants (*wdpks1*Δ) in mice and documented a dramatic decrease in mortality when the mice were infected with the melanin-deficient strains carrying *wdpks1*Δ compared to those infected with wild-type strains [93]. Infections with melanin-deficient strains resulted in a survival rate of 90–100%. In contrast, infections with the wild-type *E. dermatitidis* strain and the *wdpks1*Δ complemented strains resulted in a 20% survival rate at day 13 of the experiment [93]. In another study examining the virulence of wild-type *E. dermatitidis* compared to melanin-deficient mutants in mice, Dixon *et al*. detected a reduced mortality rate in the Mel3-infected group (0% at 21 d after infection) compared to those infected with wild-type strains (100% at day 6 after infection) [92]. However, the brains of the mice were severely affected by infections with both strains. Whereas the wild-type strain showed invasive hyphal growth, associated with acute and fatal infections, the mutant strain did not show these forms of growth [92]. In contrast, a lack of melanin production does not result in higher susceptibility to anti-infective agents [123].

Carotenoids are, alongside melanin, an important group of pigments synthesized by black-yeasts [95]. In contrast to melanin, carotenoids do not function by the neutralization of harmful oxidants. Instead, they act to shield sensitive molecules or organelles [19,104]. Environmental stress, e.g. osmotic and oxidative stress, leads to increased production of the pigment, thus contributing to cell membrane stability of the carotenogenic yeast [124,125].

In addition to melanin and carotenoids, chitin synthesis also contributes to the virulence of *E. dermatitidis* [100]. Chitin is part of the fungal cell wall with higher amounts in filamentous than in yeast-like forms [19,126]. Wang *et al*. deduced that *WdCHS2* encodes a class I chitin synthase. It is not essential; however, it is responsible for most of the chitin synthase zymogenic activity as detected *in vitro*. Mutants with double or triple mutations of the *WdCHS2* were shown to be less virulent in *in vivo* experiments compared to the wild-type. In contrast, strains with one mutation in the *WdCHS2* are as virulent as wild-type strains [100]. Chitin synthase class V, also present in *E. dermatitidis* and encoded by *WdChs5p*, is a chitin synthase which is known to be responsible for growth at extreme temperatures [98].

### Adhesion and biofilm formation

Biofilm-associated infections with fungi are often refractory to targeted treatment due to increased resistance to antifungal drugs, resulting in recurrent and chronic infections [127]. The embedded mode of life in the self-produced extracellular matrix within the biofilm provides the cells further protection against molecules of the host organism and against anti-infective agents [127]. Thus, biofilm formation contributes to the pathogenic potential of fungi.

In response to certain environmental surroundings, the majority of *Exophiala* spp. are known to exhibit strong morphological plasticity, e.g. the switch from yeast to the filamentous, hydrophobic state [19]. One other example is the cell–cell adherence, forming balls of conidia. These balls of conidia are essential for the initialization of adhesion to target cells, thus may further explain the neurotropic character of *E. dermatitidis* in parts [19].

Biofilm formation of *E. dermatitidis* in several environmental settings has been reported, e.g. at water outlet fittings in indoor environments, such as water taps and shower heads [128] or on rubber seals in dishwashers in multispecies consortia with bacteria. In the latter, Zupančič *et al*. reported also on cross-kingdom synergy of bacteria and *E. dermatitidis* within biofilms [129].

**Figure 5.**
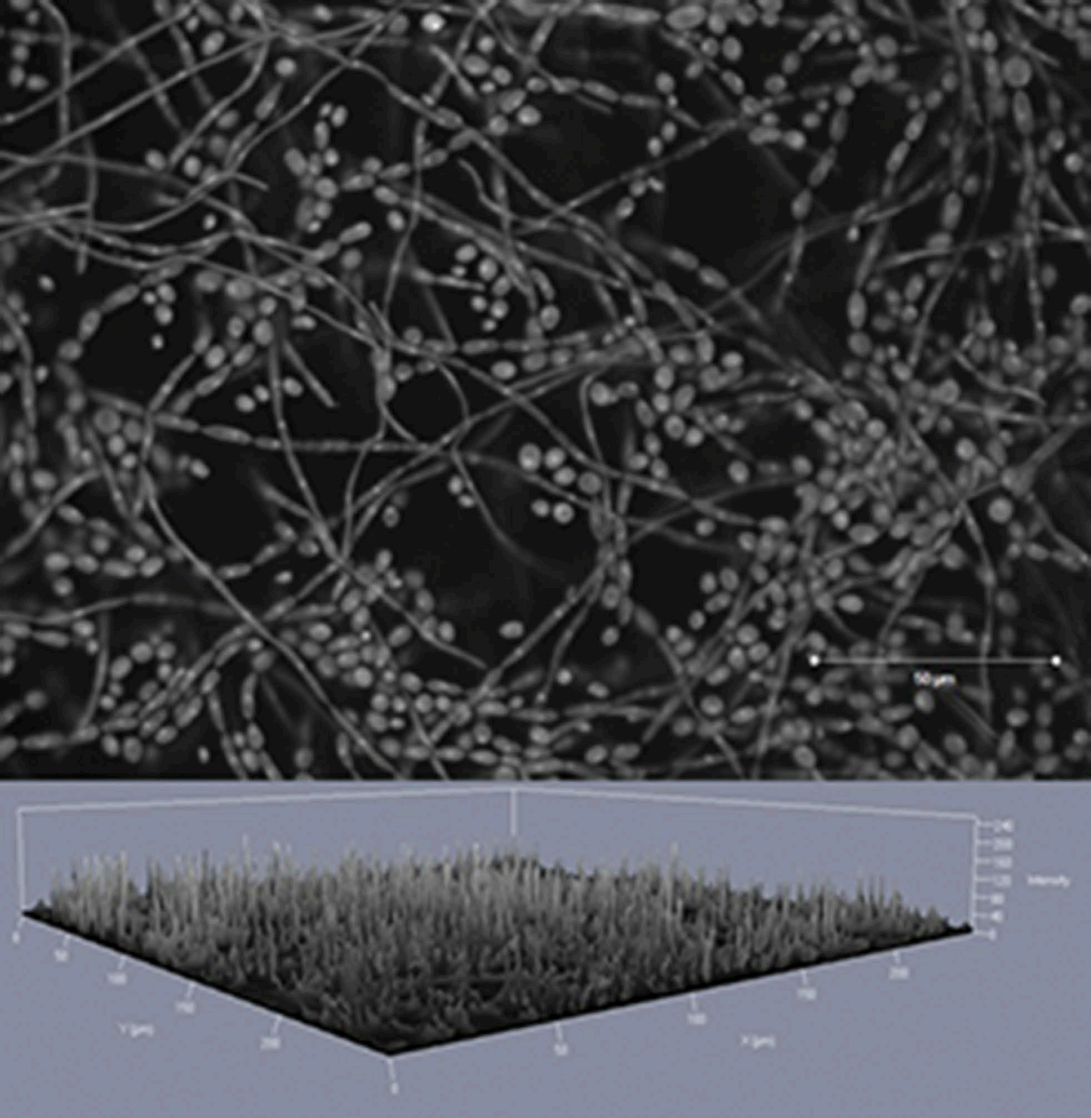
*E. dermatitidis* (CBS 116372) biofilm after 48 h of incubation at 35°C, fixation in methanol and staining by acridine orange in a confocal laser scan microscopy. Taken from: Kirchhoff et al., 2017 [130].

Next to biofilms formed in the man-made environment, the biofilm formation capabilities of clinical *E. dermatitidis* isolates have been investigated in two recent studies (Figure 5) [130,131]. Sav *et al*. identified the biofilm formation capabilities in 15% of environmental (total n = 137) and 29% of clinical isolates (total n = 7) [97]. In a study including 58 *E. dermatitidis* isolates from both, environmental and clinical sources, the biofilm formation capabilities of the fungus were demonstrated *in vitro* using a crystal violet stain based assay [130]. Biofilm formation was detected to be significantly higher after a formation period of 48 h compared to 24 h. This might be due to the slow-growing character of black yeasts. Furthermore, a difference in biomass involved in biofilm was detected for the invasive isolates from Asian patients [130].

Two studies showed that biofilm of *E. dermatitidis* displays a greater degree of resistance compared to planktonic *E. dermatitidis* cells as detected by MIC experiments [130,132].

## Susceptibility and resistance

Several studies investigated resistance and susceptibility of both environmental and clinical isolates to various anti-infective agents, using planktonic as well as sessile cells.

Currently there are no available standardized broth microdilution methodologies or validated MIC breakpoints for *in vitro* resistance testing for *E. dermatitidis*. However, numerous studies dealing with susceptibility patterns of the black yeast-like fungus exist [21,29,41,45,123,130,132–140].

Voriconazole, itraconazole, and posaconazole were shown to be active against *E. dermatitidis* as reported by Gao *et al*. [132] (Table 3). In a study from Nweze and Ezute from 2010, 16 *E. dermatitidis* isolates from stool samples in Nigeria were analyzed (CLSI) [133]. Almost all tested strains were susceptible to amphotericin B (MIC = 0.25–2 µg/mL), 5-fluorcytosine (MIC = 0.25–1 µg/mL), itraconazole (MIC = 0.25–8 µg/mL), fluconazole (MIC = 8–64 µg/mL) and voriconazole (MIC = 0.25–1 µg/mL) [133]. Duarte *et al*. analyzed a set of 43 environmental *E. dermatitidis* isolates for their susceptibility to itraconazole (MIC_50_ = 0.06 µg/mL), voriconazole (MIC_50_ = 0.06 µg(mL), flucytosine (MIC_50_ = 1 µg(mL), terbinafine (MIC_50_ = 0.015 µg/mL) and amphotericin B (MIC_50_ = 1 µg/mL) (CLSI). The MIC ranges were similar to those detected in other studies analyzing clinical isolates (Table 3) [136]. This is comparable to the findings of Deng *et al*., stating high *in vitro* activity of both terbinafine (MIC_50_ = 0.031–0.5 µg/mL) and amphotericin B (MIC_50_ = 0.125–4 µg/mL) [135]. Badali *et al*. tested eight antifungal agents for their activity against both clinical and environmental isolates of *E. dermatitidis* (CLSI). Posaconazole had the highest activity against the fungus, with a MIC_50_ of 0.063 µg/mL (CLSI) [134]. In contrast, the echinocandins caspofungin (MIC_50 =_ 4 µg/mL) and anidulafungin (MIC_50 =_ 2 µg/mL) demonstrated weak activity against *E. dermatitidis* [134]. A MIC of 8 µg/mL micafungin against three different clinical isolates of *E. dermatitidis* was detected, indicating also a weak activity of micafungin against the fungus (EUCAST) [130]. Supporting this finding, a case report in 2018 described an *E. dermatitidis* breakthrough infection during prophylactic therapy with micafungin [141]. Conversely, micafungin, when applied in lower dosages, showed antibiofilm activity. This activity was observed both when micafungin was added prophylactically to cultures, as well as when applied to mature biofilm [130]. In general, caspofungin showed a broad range of MICs against *E. dermatitidis*, varying greatly within and between studies, as summarized in Table 3 [29,45,134].

**Table 3.** MIC ranges of several drugs (No. of isolates) against *E. dermatitidis* isolates from various origins. AMB = amphotericin B, ANI = anidulafungin, CAS = caspofungin, COL = colistin, FLC = fluconazole, ISC = isavuconazole, ITC = itraconazole, MICA = micafungin, POS = posaconazole, TBF = terbinafine, VRC = voriconazole, 5-FC = fluorcytosine.

Strain origin	MIC ranges in μg/mL (No. of isolates) for													References
	AMB	5-FC	FLC	ISC	ITC	POS	VRC	ANI	CAS	MICA	TBF	COL	FK506	
**Clinical**	0.094–4 (172)	0.012-≥ 100 (70)	0.25-≥100 (58)	0.031–1 (66)	0.016–8 (182)	<0.002–0.25 (162)	0.004–16 (188)	0.25 -≥32 (3)	0.008- ≥32 (4)	8->32 (6)	≤0.01 (11)	64-≥512 (3)	>16 (16)	[22,30, 43, 47,123, 130, 132–135, 138, 140]
**Environ.**	0.12–4 (112)	0.12-≥64 (43)	2->64 (109)	0.125–1 (15)	≤0.015–2 (109)	0.016–2 (66)	0.015–2 (112)	0.25–8 (1)	2–16 (1)	-	-	≤0.003–4 (97)	-	[134–137]

FK506 (also known as tacrolimus) did not show an inhibitory effect (16 µg/mL) against planktonic *E. dermatitidis* and had MICs above 64 µg/mL against *E. dermatitidis* biofilm (CLSI) [134]. However, when analyzed in combination with itraconazole, posaconazole and voriconazole, favorable synergistic effects were demonstrated for the vast majority of a total of 16 tested strains when grown in planktonic cultures [132]. Against *E. dermatitidis* biofilm cells, the combination of azoles with FK506 also decreased the azole concentration necessary for inhibition [132].

Overall, no significant differences in antifungal activity were observed, regardless of source (environmental or clinical) [135] and type of isolate (invasive or mucocutaneous) [134]. The most promising anti-infective effects against *E. dermatitidis* were reported from the triazoles voriconazole and posaconazole [142]. Schwarz *et al*. also highlighted the possibility of treatment with posaconazole for *E. dermatitidis* infections in CF [143]. Patients suffering from CF have been shown to benefit from a combined therapy of posaconazole with amphotericin B. Recurrent *E. dermatitidis* infections in CF are recommended to be treated with posaconazole [143]. *In vitro*, amphotericin B showed additional synergistic activity in combination with terbinafine, and has been recommended as a possible treatment strategy in chromoblastomycosis caused by *E. dermatitidis* [135].

Novel antifungals agents against *E. dermatitidis* have also been tested. Isavuconazole showed *in vitro* activity against two *E. dermatitidis* reference strains [144]. In contrast, the novel dihydroorotate dehydrogenase inhibitor olorofim (F901318) had no effect (MIC > 4µg/mL) on *E. dermatitidis* [145]. Evaluating other compounds with antifungal activity against black yeasts was also performed in the last years. Fungicidal activity of N-Chlorotaurine against *E. dermatitidis* was recently reported when added to both cystic fibrosis sputum medium (simulating the CF lung environment) and standard medium in a concentration of 1–0.1% [146]. Alongside anti-mycotic agents, the success of adjunctive IFN-γ therapy in a CF patient with progressive respiratory morbidity secondary to *E. dermatitidis* infection was reported [147]. The antibiotic colistin which is also used for inhalation therapy in CF patients exhibited antifungal activity [148] and was able to reduce mature biofilm [130]. Future research in developing novel anti-infective agents for the treatment of *E. dermatitidis* infection is warranted.

In CNS infections with *E. dermatitidis*, therapeutic studies suggest the complete excision of the brain abscess. This results in better outcomes compared to partial excision or antifungal therapy [139]. However, if therapy with antifungals is preferred, the azoles voriconazole and posaconazole are recommended in the ESCMID/ECMM joint clinical guidelines for phaeohyphomycosis. Voriconazole is able to penetrate into brain tissue, providing clinical improvements in patients with invasive CNS mycosis. In contrast, therapy with amphotericin B alone is known to have a poor outcome in this patient group. Combination therapy of a triazole with fluorcytosine is a possible first-line therapy when surgery is not feasible [139]. The combination of amphotericin B and fluorcytosine is also a useful treatment regime for fungal infections of the central nervous system (e.g. cryptococcal meningitis). This combination showed a high degree of synergism *in vitro* against three tested *E. dermatitidis* isolates [149]. This therapeutic approach with surgical resection was successful in a patient with chronic granulomatous disease and progressive pulmonary and CNS infection with *E. dermatitidis* [150]. More reports of patient cases or case series are needed to gain more evidence.

## Conclusion

*E. dermatitidis* is an emerging opportunistic pathogen among the ever-increasing numbers of immunocompromised hosts. Several virulence factors like melanization, dimorphism, and biofilm formation have been studied and evaluated in *in vitro* and animal models. Understanding the mechanisms of transmission, pathogenicity and resistance will be essential for developing new strategies for better diagnosis and treatment of serious *E. dermatitidis* infections. In addition, it will be important to explore pathogen-specific adaptation mechanisms to the host. Studying the immune response associated with *Exophiala dermatitidis* infection is also a cornerstone to future research. Furthermore, the development of standardized methods for the detection and identification of *E. dermatitidis*, as well as the definition of species-specific breakpoints of antifungals are currently under study.
